# Influence of Grapheme and Syllable Learning on Handwriting Output of Chinese Characters in Children With Dictation Difficulties

**DOI:** 10.3389/fpsyg.2018.01671

**Published:** 2018-10-01

**Authors:** Yaqian Tan, Xiangping Liu

**Affiliations:** ^1^School of Psychology, Beijing Normal University, Beijing, China; ^2^Department of Applied Social Sciences, City University of Hong Kong, Hong Kong, Hong Kong; ^3^Beijing Key Laboratory of Applied Experimental Psychology, School of Psychology, Beijing Normal University, Beijing, China

**Keywords:** Chinese dictation difficulties, grapheme and syllable binding, writing, Chinese character, Chinese character learning

## Abstract

Studies on Chinese dictation difficulties have focused on visual processing and phonological processing. In recent decades, attention has shifted to the ability to bind visual and auditory information. However, such studies are scarce and rarely focus on how this ability influences children’s learning and writing of Chinese characters. In this study, a group of children with Chinese dictation difficulties and a control group without such difficulties were instructed to learn rarely used Chinese characters under three learning modes: grapheme learning, syllable learning, and grapheme-syllable learning. Participants’ learning time and writing accuracy were recorded under each learning mode. Findings showed that under the grapheme learning mode, learning time and writing accuracy failed to differ significantly between the two groups. However, under the grapheme-syllable learning mode, the writing accuracy of children with dictation difficulties was significantly lower than controls. These findings, taken together, suggested that for children with dictation difficulties, learning graphemes and syllables at the same time did not improve their writing performance as much as the controls. Under the syllable learning mode, learning time and writing accuracy failed to differ significantly between the two groups. The findings contributed to a better understanding of the Chinese dictation difficulties.

## Introduction

**FIGURE 1:**
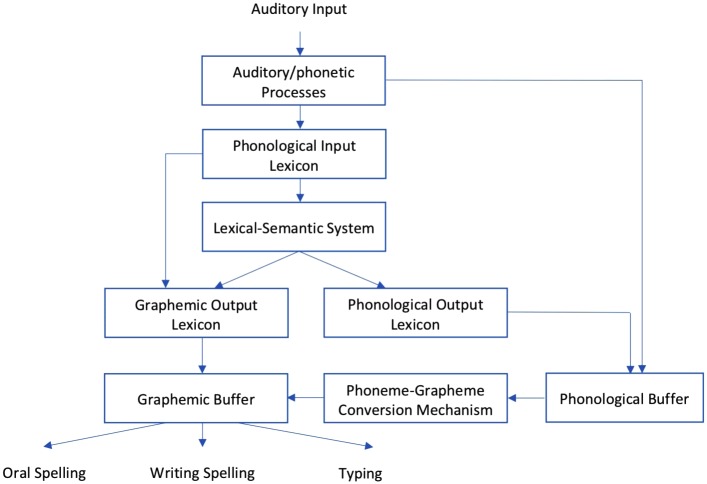
Dual-route model of spelling process. From Caramazza et al. (1987), Miceli (1989), Tainturier and Rapp (2001), Houghton and Zorzi (2003), Rapcsak et al. (2007), and Liu et al. (2008).

Written spelling is important for school-age children. In school, not being able to write puts a child in a disadvantageous position, because writing is a primary means of organizing, exploring, and developing one’s ideas as well as communicating with teachers and other classmates via assignments, exercises, and examinations. Spelling is more difficult than reading (Bosman and Van Orden, 1997), but research on spelling and writing has received very little attention, when compared with studies on other cognitive functions such as reading (Miceli, 1989). A dual-route model has emerged to explain the spelling process (Caramazza et al., 1987; Miceli, 1989; Tainturier and Rapp, 2001; Houghton and Zorzi, 2003; Rapcsak et al., 2007; Liu et al., 2008). This model assumed that spelling is accomplished through two distinct processing procedures for computing graphemic representations for familiar and novel words or non-words, namely the *lexical and non-lexical routes* (**Figure 1**). Processing of familiar words relies on the lexical route, wherein an auditory input activates all relevant phonological representations in the mind; later, semantic representations in the lexical-semantic system are further activated. The phonological and semantic representations together activate the specific graphemic representations and, finally, lead to the spelling output. The non-lexical route processes novel words or non-words, the auditory input transfers into graphemes according to the phoneme to grapheme conversion (PGC) mechanism.

Dictation is one of the most basic forms of spelling and writing. The process of dictation involves the central processes of retrieving, assembling, and selecting an orthographic representation (which is called *spelling*) as well as peripheral processes of the output and execution of orthographic codes (which is called *writing*; Delattre et al., 2006). Most studies on Chinese writing and spelling are about dictation (Zhang et al., 2006; Pan et al., 2010; Yang et al., 2010a,b); however, relevant research remains scarce. To date, no clear definition of Chinese dictation difficulties exists, and no model is available to elucidate how writing develops in the Chinese language.

Previous studies on Chinese dictation difficulties have focused on visual processing and phonological processing. Visual processing is defined as “the ability to process two-dimensional visual representations, such as shapes, dots, and lines” (Li et al., 2010), and it, supposedly, plays an important role in learning to read Chinese (Luo et al., 2013). The orthographic form of alphabetic writing systems is a conventional representation of the pronunciation of a word (Bisani and Ney, 2008), characterized by grapheme-phoneme correspondence rules (Gibson et al., 1962; Landerl et al., 1997). However, as ideography, the Chinese language is a “deep” orthography with little correspondence between graphemes and syllables (Hu and Catts, 1998; He et al., 2016). Chinese characters correspond to morphemes rather than to individual phonemic units of spoken language (Feldman and Siok, 1999). Furthermore, when compared with alphabetic languages that use a limited number of letters to produce all words (Li et al., 2010), Chinese characters consist of extensive and highly complicated visual information. Each Chinese character is a combination of strokes, components, or radicals (Pak et al., 2005), and the variety in shape, size, spacing, placement of stokes, components, and radicals increases the difficulty in reading and writing. For example, 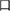/kou3/(mouth) is a frequently used radical in Chinese characters, but its size and placement vary greatly across its compound characters, such as 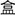/he2/(box), 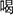/he1/(drink), and 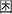/kun3/(sleepy). Moreover, the Chinese language contains many near-homographs, such as 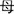/mu3/ (mother) and 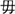/wu4/ (no), rendering the identification and writing of Chinese characters much more difficult. The low level of correspondence between graphemes and syllables, coupled with the visual complexity of Chinese characters, has led to the hypothesis that visual processing is critical for Chinese dictation and reading (Zhang, 2006; Li et al., 2010; Mao, 2015; Tan et al., 2016). Several studies have focused on visual discrimination and visual memory abilities. Evidence from Chinese reading showed that visual skills correlated significantly with word recognition among kindergarteners (Li et al., 2010). Studies on Chinese dictation indicated that visual discrimination was significantly weaker in children with dictation difficulties than in other children (Zhang, 2006; Mao, 2015), but their visual memory and global processing of visual information were at the same level as the other children (Mao, 2015). However, another study found that children with Chinese dictation difficulties were impaired in global processing of visual information (Tan et al., 2016).

In addition to visual processing, phonological processing has also been studied. Phonological processing refers to the “use of phonological information in processing written and oral language” (Wagner and Torgesen, 1987), and it involves phonological awareness, phonological memory, and phonological naming (Wagner et al., 1997). For alphabetic languages, phonological processing tasks tend to associate highly with word reading (Hulme et al., 2002). Chinese language, by contrast, belongs to the morphosyllabic system, with one character corresponding to a single syllable that contains one consonant and vowel(s). In general, Chinese characters have a relatively simple syllabic structure, as compared with alphabetic languages. The relationship between phonological awareness and reading is much weaker in the Chinese language than in alphabetic languages (Tan et al., 2005). However, other studies have still identified a strong relationship between phonological processing skills and reading ability for Chinese characters (Hu and Catts, 1998; Ho and Lai, 1999; Chan and Siegel, 2001; Shu et al., 2008; Li et al., 2010), and this is possibly attributable to the use of *pinyin* (romanization of Chinese) (Shu et al., 2008). Studies of Chinese reading and dictation have focused on phonological awareness and phonological naming (Dai et al., 2016). For example, Li et al.’s (2010) study showed that phonological awareness and rapid number naming were consistently associated with Chinese character reading among kindergarteners and primary school students. Shu et al. (2008) found that syllable awareness, tone awareness, and rapid automatized naming were strongly associated with character recognition among preschool children. Studies on phonological memory have mainly been conducted in Hong Kong and Taiwan, where traditional Chinese scripts are used. For example, So and Siegel (1997) found a moderate correlation between phonological working memory and character recognition in primary school students. Another study demonstrated that children with both writing and reading disabilities performed poorly in both word and non-word repetition, when compared with normally developed children (Ho et al., 2000). In the field of dictation difficulties, studies on phonological processing are rare. A study revealed that children with Chinese dictation difficulties exhibited weaker phonological awareness than normally developed children (Zhang, 2006).

Apart from visual and phonological processing, increasing attention has been paid to the ability to bind visual and auditory information (Yang et al., 2009a, 2010a; Liu et al., 2014; Dai et al., 2017; Ning et al., 2017). The ability to generate detailed connections between visual and phonological forms of words is the foundation for building a consolidated sight word vocabulary (Ehri, 2005) and for the development of reading and spelling skills (Moll et al., 2016). As there is little correspondence between the graphemes and syllables in Chinese characters, their interconnection is more arbitrary. Success in building connections between the grapheme and syllable seems to be more difficult than alphabetic scripts. Mapping orthography to syllable is important in learning to read Chinese (Zhang et al., 2016), and it is a particularly important skill to distinguish between children with and without Chinese dyslexia (Li et al., 2009). However, in regard to Chinese dictation difficulties, only a few studies have focused on the visual-auditory binding ability. These studies indicated that children with dictation difficulties demonstrated a deficit in binding visual and auditory information (Zhang et al., 2006; Yang et al., 2009a; Zhong et al., 2011; Liu et al., 2014), and this deficit was related to their failure to write Chinese characters (Yang et al., 2009a). Further studies revealed that children with dictation difficulties could form visual-auditory associations when the visual stimulus or task was simple. As the complexity of a visual stimulus or task increased, their visual-auditory association performance became poorer when compared with normally developed children; however, the complexity of an auditory stimulus failed to influence their performance (Yang et al., 2010a; Liu et al., 2014). These findings indicated that their deficit in visual-auditory binding was connected to deficits in cross-modal association or visual/orthographic processing. However, other studies showed contradictory results. For example, one study found that children with dictation difficulties also performed more poorly than other children in binding two auditory stimuli, while their ability to bind two visual stimuli was at the same level as the other children (Liu et al., 2014; Dai et al., 2017). Thus, the influence of visual and auditory characteristics in the formation of associations remains unclear.

Although deficits in visual-auditory association were largely supported, the existing studies have only focused on the association between non-verbal stimuli, namely irregular figures and nonsense sounds (Liu et al., 2014; Dai et al., 2017). According to the dual coding theory (DCT, Paivio, 1990), verbal and non-verbal representations are processed and stored in two theoretically distinct systems: the verbal system, which contains visual, auditory, articulatory, and other modality-specific verbal codes; and the non-verbal system, which includes images for shapes, environmental sounds, actions, and other non-linguistic objects and events (Clark and Paivio, 1991). Thus, the questions of how the deficiency in non-verbal visual-auditory binding ability affects Chinese character learning and how these findings can be applied to existing spelling models remain unexplored. Moreover, these studies used the paired association or change detection paradigm to measure the association ability, but these tasks only informed about the processing outcome (Moll et al., 2016); what happens during the learning process is unknown.

To fill these research gaps, the present study examined the visual-auditory binding ability of children with Chinese dictation difficulties at the verbal level. By manipulating learning process, we explored how grapheme learning, syllable learning, and grapheme-syllable learning of Chinese characters influence their writing output and whether children with dictation difficulties perform differently when compared with those without the difficulties. We expected that the writing performance of children with dictation difficulties would differ significantly from children without the difficulties under grapheme learning and syllable learning. We also expected that children with dictation difficulties would benefit from grapheme-syllable learning as much as children without the difficulties, when compared with grapheme learning only.

## Materials and Methods

### Participants

Participants (aged 8–12 years, grades 3–6) were recruited from one primary school in Beijing. Children with dictation difficulties demonstrated normal reading performance and lagging dictation performance; importantly, they often could read but could not write Chinese characters that were learnt previously. Based on these typical manifestations, the criteria for screening dictation difficulties mainly included the following: (a) inability to write but could read learned Chinese characters; (b) having Chinese language scores lower than the average; (c) having math scores higher than the average; (d) showing no symptom of attention deficit; (e) showing normal to above average reading ability; and (f) showing higher than average discrepancy between reading and dictation abilities (Yang et al., 2010a,b; Mao and Liu, 2012; Tan et al., 2016). The reading ability was operationalized as the reading accuracy in the reading test, and the discrepancy between reading and dictation abilities was operationalized as the ratio of the number of Chinese characters read correctly in the reading test, but wrongly written in the dictation test, to the number of Chinese characters read correctly in the reading test (Mao and Liu, 2012; Tan et al., 2016).

Following these criteria, we first invited the head teacher or the Chinese teacher of each class to provide a name list of children meeting the criteria (a), (b), (c), and (d). Meanwhile, chronologically, age-matched children showing no signs of dictation difficulties were selected. The dictation test, the reading test, and the Raven’s Standard Progressive Matrices-Chinese Revised (R’SPM-CR) test were administered to all the potential participants.

#### Dictation and Reading Tests Developed by Researchers

Following previous studies (Yang et al., 2010a,b; Mao and Liu, 2012; Tan et al., 2016), the dictation and reading tests were developed for this study and were used only for the selection of participants. The two tests consisted of Chinese characters from the students’ Chinese textbook. The Chinese textbook for each grade had two lists of characters attached at the end. Normally, after one semester, students should be able to read the characters of the first list, and read and write the characters of the second list. All the characters chosen for the reading and dictation tests were from the second list. For each grade, the characters included in the dictation and reading tests were identical.

#### Raven’s Standard Progressive Matrices-Chinese Revised (R’SPM-CR)

The R’SPM test was developed by Raven (1958) and Raven et al. (1998) to measure non-verbal intelligence. The R’SPM test was revised, and the norms were established in China (Zhang and Wang, 1985, 1989). The test included 60 items that were divided into five sets, which were constructed to become progressively more difficult while moving across the sets. The raw score was the sum of all items, with a range from 0 to 60, and was transformed to the percentile rank according to the age group. The R’SPM-CR had a split-half correlate coefficient of 0.95 and test-retest reliability of 0.82 (Zhang and Wang, 1989). Its criterion-related validity with Chinese version of Wechsler tests was 0.71 (Zhang and Wang, 1989).

The dictation test and R’SPM-CR test were administered first, and the reading test was administered 2 days later. Participants with R’SPM-CR test percentile rank lower than 25% were eliminated, 48 participants were finally selected in this study. The group with dictation difficulties consisted of 25 participants (18 boys), seven of whom were from Grade 3, six from Grade 4, four from Grade 5, and eight from Grade 6. The control group consisted of 23 participants (12 boys), seven of whom were from Grade 3, six from Grade 4, four from Grade 5, and six from Grade 6. All participants were native *Putonghua* speakers and had normal or corrected-to-normal vision. The descriptive characteristics of the two groups are presented in **Table 1**. For the study, signed informed consent from all participants and their guardians was obtained prior to the experiment. The experiment was approved by the Academic Committee of the School of Psychology, Beijing Normal University, China.

**Table 1:** Descriptive characteristics of dictation difficulties and control group.

	Dictation difficulties		Control		
	*M*	*SD*	*M*	*SD*	*t*-value
Age (years)	10.380	1.317	10.413	1.379	-0.085
R’SPM-CR (percentile)	59.100	21.236	60.652	18.282	-0.270
Reading accuracy	0.822	0.078	0.845	0.028	-1.390
Dictation accuracy	0.580	0.114	0.720	0.877	-4.749^∗∗∗^
Discrepancy between reading and dictation	0.518	0.156	0.330	0.108	4.868^∗∗∗^

*R’SPM-CR = Raven’s Standard Progressive Matrices-Chinese Revised.*

*^∗∗∗^*p* < 0.001*.

### Materials

To control for participants’ familiarity with Chinese characters, rarely used Chinese characters (i.e., those rarely seen or used in everyday life) were chosen as materials for the present study. For the study, 76 rarely used Chinese characters were chosen (**Table 2**); all of them were 2-component characters, with 38 left-right structured and 38 top-down structured, and all the chosen characters had 4–8 strokes. These characters met the orthographic regularity. The average frequency of these Chinese characters was 0.02/100,000 (lower than 0.55/100,000 per character), according to the Media Language Corpus (MLC)^1^. Additionally, 20 characters were in Tone 1 (high level tone), 20 in Tone 2 (low rising tone), 12 in Tone 3 (falling-rising tone), and 24 in Tone 4 (high falling tone). Character pronunciations were not inferable from their phonetic components. Both the grapheme and syllable of these characters were used in the experiment. Pronunciation of these characters was read in *Putonghua* by a woman who had obtained a certificate of the first level, class B in the *Putonghua* level test. The experiment used 10 characters in the practice block and the remaining 66 in the experiment blocks.

**Table 2:** Example of experimental stimuli and their properties.

Structure	Character frequency	Shape	Pronunciation	Strokes
Left-right	0.00/100,000	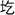	/ge1/	6
	0.05/100,000	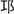	/qiong2/	5
	0.00/100,000	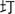	/ting3/	5
	0.00/100,000	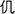	/zhang4/	4
Top-down	0.00/100,000	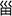	/zai1/	8
	0.00/100,000	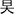	/tai2/	7
	0.00/100,000	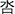	/qi3/	7
	0.00/100,000	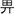	/bi4/	8

### Procedure

Participants completed the experiment individually in a quiet computer classroom in their school. The experiment was run using E-prime Version 1.1 on computers with a 15-inch screen and Windows 8 operating system. Participants sat in front of the computers, with their eyes leveled with the screen, wearing earphones. Procedure and requirements were introduced to participants before they entered the practice block. After 10 practice trials, participants were asked to confirm whether they fully understood the requirements and procedure of the experiment. If so, they could enter the experiment; otherwise, requirements and procedure were introduced again in a more detailed way. Participants had to go through the practice block again until they were eligible to take up the experiment.

**FIGURE 2:**
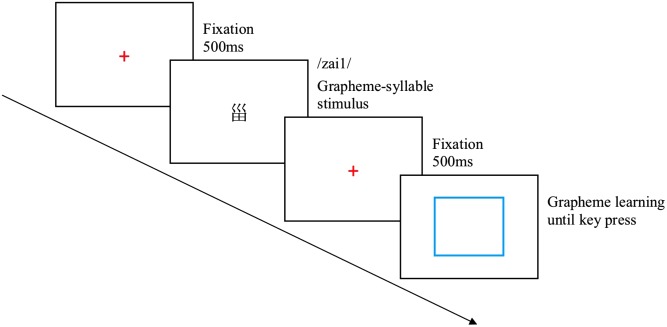
Presentation format of each trial under the grapheme learning mode. Each trial applied the presentation format as follows: fixation, grapheme-syllable stimulus, fixation, white screen. Participants were required to press “J” on the keyboard and write down the grapheme of the stimulus on paper.

The experiment included three blocks for grapheme learning, syllable learning, and grapheme-syllable learning. Each block consisted of 22 trials. In each trial, a fixation point was presented for 500 ms in the center of the screen, followed by a grapheme-syllable stimulus for 3000 ms. The grapheme of the stimulus was displayed in the center of the screen. Meanwhile, pronunciation of its syllable was broadcasted via the earphone and was repeated three times. Following the stimulus, a fixation point was presented in the screen center for 500 ms. When the fixation point disappeared, participants were required to learn the stimulus with grapheme learning mode, syllable learning mode, or grapheme-syllable learning mode, depending on the particular block they were in. For the grapheme learning block (**Figure 2**), the fixation point was followed by a white screen with a blue square frame in the screen center. Participants needed to recall the grapheme of the stimulus within the blue square frame. To avoid learning the syllable of the stimulus, participants had to orally repeat “/yi1/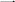
(one), /er4/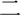
(two), /yi1/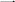
(one), /er4/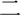
(two)” loudly during their learning. For the syllable learning block, the fixation point was followed by a short paragraph of a story written in Chinese that was presented inside a blue square frame in the screen center. Participants needed to orally repeat the pronunciation of the stimulus. To avoid learning the grapheme of the stimulus, participants had to read the story on the screen silently during their learning. For the grapheme-syllable learning mode, the fixation point was followed by a white screen with a blue square frame in the screen center. Participants needed to recall the grapheme of the stimulus within the blue square frame and had to orally repeat the syllable of the stimulus at the same time. When participants thought that they fully remembered the stimulus, they pressed the “J” on the keyboard and wrote the grapheme of the learned stimulus on an answer sheet and, then, pressed the “F” on the keyboard to enter the next trial. All participants were required to complete all of the three learning blocks. The order in which the participants completed the blocks was counterbalanced.

### Data Analysis

Two dependent variables were recorded: learning time and writing accuracy. Learning time was the interval between the disappearance of the fixation following the stimulus and the participants’ pressing of “J” on the keyboard in each trial, which was automatically recorded by E-prime. Writing accuracy was calculated upon the completion of the experiment.

The effects of the learning modes and the groups on learning time and writing accuracy were explored in two analyses of variance (ANOVAs), with the group (dictation difficulties vs. controls) as between-subjects factor and the learning mode (grapheme learning vs. syllable learning vs. grapheme-syllable learning) as within-subjects factor. Besides, the difference in learning time between the grapheme-syllable and syllable learning modes and between the grapheme-syllable and grapheme learning modes were calculated for each group, and independent-sample *t*-tests were applied to make direct comparisons between the two groups on these differences.

## Results

### Learning Time

The mean scores and standard deviations of learning time across three learning modes for both dictation difficulties and control groups are presented in **Table 3**.

**Table 3:** Mean (SD) learning time (second) on three learning modes.

	Dictation difficulties		Control	
	*M*	*SD*	*M*	*SD*
Grapheme learning	6.219	3.703	8.015	4.487
Syllable learning	7.561	2.958	7.869	4.099
Grapheme-syllable learning	6.521	2.984	5.355	3.211

The ANOVA revealed no significant main effect of the group [*F*(1,46) = 0.172, *p* = 0.680, η^2^ = 0.004]. The learning mode showed a significant main effect, [*F*(2,92) = 4.223, *p* = 0.018, η^2^ = 0.084]. Interaction between the group and the learning mode was marginally significant [*F*(2,92) = 2.831, *p* = 0.064, η^2^ = 0.058]. Further analysis suggested that learning time failed to differ significantly between the two groups across the three learning modes. However, for control group, learning time under the grapheme-syllable learning mode was significantly shorter than those under the grapheme learning mode (*p* = 0.034) and syllable learning mode (*p* = 0.008). Nevertheless, for the group with dictation difficulties, no significant difference was found in learning time across the three learning modes.

The mean scores and standard deviations of the difference in learning time between learning modes for both dictation difficulties and control groups are presented in **Table 4**.

**Table 4:** Mean (SD) difference in learning time (seconds) between learning modes of two groups.

	Dictation difficulties		Control	
	*M*	*SD*	*M*	*SD*
Difference between the grapheme-syllable and syllable learning modes	-1.040	2.627	-2.514	4.769
Difference between the grapheme-syllable and grapheme learning modes	0.302	4.477	-2.660	5.204

Independent sample *t*-tests revealed that for the difference in learning time between the grapheme-syllable and syllable learning modes, the two groups failed to show a significant difference (*t* = 1.310, *p* = 0.199, Cohen’s *d* = 0.38). However, for the difference in learning time between the grapheme-syllable and the grapheme learning modes, the two groups showed a significant level of difference (*t* = 2.118, *p* = 0.040, Cohen’s *d* = 0.60), indicating that the difference in learning time between the grapheme-syllable learning and syllable learning was significantly larger in the control group than in the group with dictation difficulties.

### Writing Accuracy

The mean scores and standard deviations of writing accuracy across the three learning modes for both dictation difficulties and control groups are presented in **Table 5**.

**Table 5:** Mean (SD) writing accuracy (proportion correct) in three learning modes.

	Dictation difficulties		Control	
	*M*	*SD*	*M*	*SD*
Grapheme learning	0.782	0.133	0.818	0.122
Syllable learning	0.647	0.175	0.704	0.172
Grapheme-syllable learning	0.742	0.126	0.883	0.108

The ANOVA revealed a significant main effect of the group [*F*(1,46) = 5.564, *p* = 0.023, η^2^ = 0.108, see **Table 5**], indicating that writing accuracy was significantly higher in the control group than in the group with dictation difficulties. The learning mode also showed a significant main effect [*F*(2,92) = 26.284, *p* = 0.000, η^2^ = 0.364]. Interaction between the group and the learning mode was also significant [*F*(2,92) = 3.569, *p* = 0.042, η^2^ = 0.072]. Further analysis indicated that there was a significant difference in the writing accuracy between the two groups under the grapheme-syllable leaning mode (*p* = 0.000).

## Discussion

In this study, we aimed to further explore the ability to bind visual and auditory information in children with Chinese dictation difficulties, which is crucial for language learning, especially for reading and writing Chinese (Zhang et al., 2016). In particular, we aimed to uncover how deficiency in this ability affects children’s learning of Chinese characters. In this study, participants were required to learn Chinese characters by different learning modes, that is, only learning the grapheme, only learning the syllable, or learning both the grapheme and the syllable. Differences in writing performance under these learning modes allowed us to investigate deeper into the deficiency in visual-auditory binding ability in children with Chinese dictation difficulties.

The findings supported our hypotheses, which were as follows. The writing performance of children with dictation difficulties would not differ significantly from children without the difficulties under grapheme learning and syllable learning, and they would not benefit from the grapheme-syllable learning as much as children without the difficulties, when compared with grapheme learning only. Accordingly, under the grapheme learning mode and syllable learning mode, learning time and writing accuracy between children with Chinese dictation difficulties and controls failed to differ significantly. However, under the grapheme-syllable learning mode, difference in writing accuracy between the two groups reached a significant level. Analysis of the difference in learning time between the grapheme-syllable learning mode and grapheme learning mode revealed that the difference in the group with Chinese dictation difficulties was significantly smaller than the control group. Thus, on the basis of grapheme learning, adding syllable learning significantly shortened learning time and improved writing accuracy in children without dictation difficulties, but such an effect was not observed in children with the difficulties. Thus, processing grapheme and syllable information simultaneously is not the preferential way for children with Chinese dictation difficulties to learn Chinese characters. These results were consistent with previous studies that were conducted using irregular figures and nonsense syllables as experimental materials to identify whether children with Chinese dictation difficulties were weaker in binding visual and auditory information than normally developed children (Zhang et al., 2006; Liu et al., 2014). Specifically, children with Chinese dictation difficulties performed more poorly than children without the difficulties in recognizing or recalling visual-auditory pairs that were learnt previously (Mao and Liu, 2012). In line with these findings, we further explored to see whether this kind of deficit applied to Chinese characters. In regard to this, we found that when children with Chinese dictation difficulties were learning new Chinese characters, learning the grapheme and syllable simultaneously failed to improve their writing performance as much as that observed in the control group.

Based on these findings, we now discuss in more detail why and how children with Chinese dictation difficulties do not benefit from learning grapheme and syllable simultaneously, as compared with only learning grapheme. One possible explanation is that, due to their deficiency in binding visual and auditory information, orthographic and phonological information in Chinese characters are loosely associated, or even separated, in their mental lexicon. As posited by the parallel distributed processing (PDP) approach (Plaut et al., 1996), lexical transformations among orthographic, phonological, and semantic representations are accomplished by interactions among orthographic, phonological, and semantic unites, as governed by the weighted connections among them (Joanisse and McClelland, 2015). Yang et al. (2008) found a positive effect of phonetic radical cues on dictation performance in children without dictation difficulties, but this effect did not appear in children with dictation difficulties when the phonetic radical shared the same vowel(s) with the whole character. Even when the phonetic radical shared the same pronunciation with the whole character, the positive effect was significantly stronger in children without the difficulties. Another study (Tan and Liu, unpublished manuscript) also demonstrated similar findings, such that the presence of homophones as hints significantly improved the dictation accuracy of normally developed children, but this improvement did not occur in children with dictation difficulties. However, since children with and without dictation difficulties showed equal reading performance in this study, the connection from orthographic to phonological information might be at the normal level for children with dictation difficulties. Hence, a potential explanation is that the weights of connections from phonological to orthographic information are weaker in children with dictation difficulties, leading to the conclusion that phonological information cannot activate orthographic representation as in the case of normally developed children.

An additional explanation lies in the possibility that children with dictation difficulties are unable to process visual and auditory information of a stimulus simultaneously. The mechanism of inter-sensory integration (e.g., sounds and visual events) relies on temporal congruence (Bertelson and Radeau, 1981; Bushara et al., 2001); the temporal binding hypothesis proposes that binding happens when neuronal discharges are synchronized (Von der Malsburg, 1995; Engel and Singer, 2001). Following this hypothesis, an association between visual and auditory information emerges with simultaneous processing. Moreover, sound stimuli enhance the neural response to and the perception of a synchronized visual event (Stein et al., 1996; Van der Burg et al., 2011). This can explain why the writing performance of normally developed children benefits from learning the grapheme and syllable simultaneously. However, children with dictation difficulties automatically prioritize the processing of visual information, with a tendency to either ignore or suppress auditory information (Yang et al., 2009b). Suppressing auditory information in turn requires greater cognitive resources when compared to merely processing visual information. With limited cognitive resources or within limited time, higher requirements for cognitive resources may further cause incomplete processing of visual information. This assumption found support in the results of this study. The ANOVA result of learning time and writing accuracy together showed that children with Chinese dictation difficulties performed more poorly in the grapheme-syllable learning than in grapheme learning, although the difference failed to reach a significant level.

Being unable to process visual and auditory information simultaneously and prioritizing visual processing may further influence the representation of Chinese characters as incomplete graphemes, rather than as combinations of grapheme and syllable, in the mental lexicon of children with dictation difficulties. The direct-image hypothesis proposes that Chinese characters are directly encoded as abstract or even concrete images (Tan and Perfetti, 1998) and are represented in graphic forms, which vividly signal its meaning (Wang, 1973). This hypothesis assumes that recognition of Chinese characters does not require phonological processing. Clinical observations revealed that when children with Chinese dictation difficulties were required to write some Chinese characters that were learnt before, they only remembered some character strokes, as manifested by their incomplete writing; that is, they could either only write some character strokes or none at all (Yang et al., 2009a).

**FIGURE 3:**
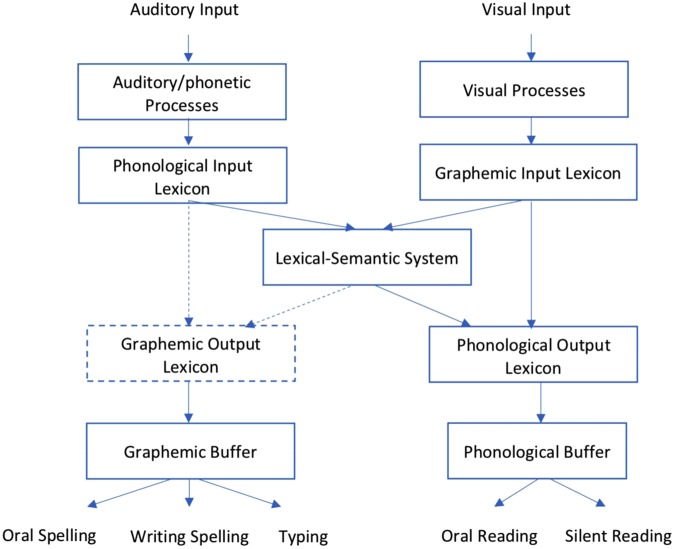
Model of writing and reading processes for Chinese dictation difficulties. Adapted from Caramazza et al. (1987), Miceli (1989), Tainturier and Rapp (2001), Han et al. (2008), Houghton and Zorzi (2003), Rapcsak et al. (2007), and Liu et al. (2008).

Based on the above discussions, we proposed a refined model of writing and reading regarding Chinese dictation difficulties (**Figure 3**) by drawing upon the dual-route models on spelling (Caramazza et al., 1987; Miceli, 1989; Tainturier and Rapp, 2001; Houghton and Zorzi, 2003; Liu et al., 2008; Rapcsak et al., 2007) and a mental lexicon model illustrating the processes of Chinese reading and writing by Han et al. (2008). The refined model included both the writing and reading processes. We do did not include the PGC rule embodied in the original spelling models, as this rule was not applicable in the Chinese language. The spelling pathway was adjusted to match our findings. The refined model represented associations from phonological input lexicon to graphemic output lexicon and those from lexical-semantic system to graphemic output lexicon by dotted-line arrows to indicate the lower than average level. This model also marked the outline border of the graphemic output lexicon by dotted-line to correspond to our hypothesis that Chinese characters are represented as graphemes in the mental lexicon of children with dictation difficulties, and, in most of the cases, these graphemic representations are incomplete. Another pathway illustrated the reading process, as children with dictation difficulties showed equal reading performance with normally developed children; we assumed that their reading pathway did not have any deficiency.

In summary, this study is the first to apply the idea of visual-auditory binding ability into the context of learning Chinese characters to explore how this ability affects the learning performance of children with Chinese dictation difficulties. Findings suggest that unlike those without the difficulties, children with Chinese dictation difficulties do not benefit from the learning grapheme and syllable information on a Chinese character simultaneously. Findings of this study, thus, contribute to a better understanding of Chinese dictation difficulties.

Finally, the limitations of the present study are noteworthy. First, although reading accuracy, dictation accuracy, and the discrepancy between reading and dictation were used to indicate participants’ reading and dictation abilities, other indicators, such as writing and spoken vocabulary sizes, could be included to improve the rigor of participant selection. Second, even though the present study controlled for some linguistic factors of the experimental materials, other phonetic and phonological characteristics, such as manner of articulation of the consonant, vowel height and intensity, should also be considered to control for potential confounding factors. Third, some low-grade participants expressed difficulty in understanding experimental requirements under the three learning modes, and they needed to go through more practice trials before entering experiment blocks, which might generate fatigue or practice effects. Fourth, the refined model we proposed was largely speculative and calls for sufficient validation. In the future, we shall conduct research with more rigid controls for the selection of participants and materials, and with simpler tasks, to validate the proposed model. Meanwhile, we can include children with Chinese developmental dyslexia in our future study for comparing the mechanisms of Chinese reading and writing.

## Author Contributions

YT designed the research, performed the experiments, analyzed the data, and wrote the manuscript. XL provided supervision and instructions during the whole process.

## Conflict of Interest Statement

The authors declare that the research was conducted in the absence of any commercial or financial relationships that could be construed as a potential conflict of interest.

## Footnotes

1 http://ling.cuc.edu.cn/RawPub/jianjie.aspx
